# Magnetisation process in the rare earth tetraborides, NdB_4_ and HoB_4_

**DOI:** 10.1038/s41598-017-18301-1

**Published:** 2018-01-10

**Authors:** D. Brunt, G. Balakrishnan, D. A. Mayoh, M. R. Lees, D. Gorbunov, N. Qureshi, O. A. Petrenko

**Affiliations:** 1 Department of Physics, University of Warwick, Coventry, CV4 7AL United Kingdom; 2 Dresden High Magnetic Field Laboratory (HLD-EMFL), Helmholtz-Zentrum Dresden-Rossendorf, 01314 Dresden, Germany; 3 Institut Laue-Langevin, 6 rue Jules Horowitz, BP 156, 28042 Grenoble, Cedex 9 France

## Abstract

A field-induced magnetisation process in the frustrated antiferromagnets is often much richer compared to the materials without competing interactions. The applied field tends to stabilise unusual spin configurations which frequently results in the appearance of magnetisation plateaux. Here we report a study into the field-induced magnetisation of the two frustrated rare earth tetraborides, HoB_4_ and NdB_4_. NdB_4_ shows a fractional magnetisation plateau occurring at *M*/*M*_sat_ ≈ $\tfrac{{\bf{1}}}{{\bf{5}}}$ before saturating in a field of 33 kOe. On cooling down to 0.5 K the temperature dependent susceptibility of NdB_4_ shows an unconventional transition where the system returns to the zero field antiferromagnetic state from a higher-temperature ferrimagnetic state. We are able to reconstruct the magnetic phase diagram of NdB_4_ from the magnetisation, susceptibility and resistivity measurements for both *H* $\parallel$ *c* and *H* ⊥ *c*. For HoB_4_, the most interesting behaviour is found at the lowest temperature of 0.5 K, where the field dependent magnetisation demonstrates a new fractional $\tfrac{{\bf{1}}}{{\bf{2}}}$-magnetisation plateau. Further insight into the relations between the exchange interactions and single ion effects is gained through high-field magnetisation measurements in both HoB_4_ and NdB_4_.

## Introduction

**Figure 1:**
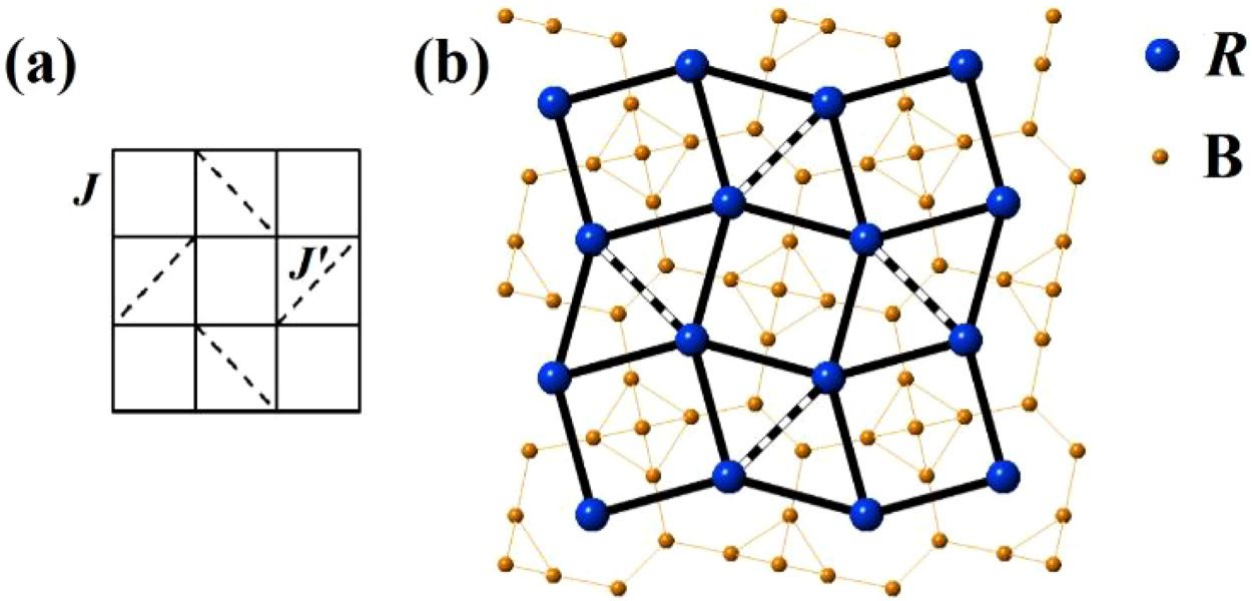
(**a**) Shastry-Sutherland lattice, (**b**) projection of *R*B_4_ structure along the tetragonal *c* axis. The *R* ions form a lattice that topologically maps to the Shastry-Sutherland lattice. Solid bonds correspond to the nearest neighbour interaction *J*, while the dashed bonds are the next nearest neighbour *J*′.

In the presence of antiferromagnetic interactions, lattices based around edge and corner sharing-triangles^1^ and tetrahedra^2^ display geometric frustration. These systems are unable to minimise the energy of the competing interactions resulting in a large ground-state degeneracy^3^. This leads to a suppression of the long-range order and often results in a diverse magnetic phase diagrams. Some notable examples include the pyrochlore^4^, garnet^5^ and kagome lattices^6^. In particular, materials such as SrCu_2_(BO_3_)_2_^7^ and the rare earth tetraboride (*R*B_4_) family, based on the frustrated Shastry-Sutherland lattice (SSL)^8^ (Fig. 1(a)) have garnered attention due to their complex phase diagrams, displaying a variety of magnetic phases in quite modest magnetic fields. The *R*B_4_ family crystallises into a tetragonal (*P*4/*mbm*) structure, where the *R* ions form a network of squares and triangles in the *ab* plane, which topologically maps to the SSL (Fig. 1(b)). The usual order amongst the *R*B_4_ is antiferromagnetic with the typical ordering being confined to the *ab* plane. One of the *R* ^3+^ ion valence electrons is given up to the conduction band making the *R*B_4_ family metallic and it is believed the RKKY interaction is important in the formation of long-range order of the magnetic moment on the rare earth ions only^9^. Plateaux with fractional values of the saturation magnetisation, *M*_sat_, are a common feature to many of the *R*B_4_ family members ranging from a $M/{M}_{{\rm{sat}}}=\tfrac{1}{2}$, corresponding to a ferrimagnetic phase in ErB_4_^10^ to a more complex striped structure displayed in TmB_4_^11^. While in some cases high magnetic fields are needed to observe the fractional plateaux^12,13^.

The initial study of the SSL considered three cases; the quantum, Ising and classical limit^8^. The classical limit in particular is well suited to describing the *R*B_4_ family due to the relatively large magnetic moment arising from the *R* ^3+^ ions. It was found that in this limit the SSL has an exact ground-state solution, with a helical arrangement of the magnetic moments. Significant theoretical work has been done more recently using Heisenberg^14,15^ and Ising^16–18^ spins in the classical limit to understand the *R*B_4_ family. Many of these studies predict the existence of fractional magnetisation plateaux. For HoB_4_ in particular it was suggested the $\tfrac{1}{3}$ plateau could be due to an *up*-*up*-*down* collinear arrangement on the Ho moments in the triangle formed by the diagonal *J*′, while another suggestion is an “umbrella” structure where the moments are tilted towards the vertical axis and are arranged at 120° relative to one another forming a spiral that propagates through the structure^14,18^. The introduction of further interactions beyond *J* and *J*′, including the RKKY interaction^19^, dipolar interaction^16^ and longer range interactions across multiple unit cells^15^, have predicted the emergence of further smaller features in the magnetisation curves. Thus gaining an insight into the exchange interactions *J* and *J*′ is an important first step to understanding the *R*B_4_ family.

In this report we have investigated the magnetisation process in two members of the *R*B_4_ family, HoB_4_ and NdB_4_. We follow the development of the magnetic order in these compounds with an applied field by measuring magnetisation and susceptibility down to *T* = 0.5 K and observe a sequence of unconventional transitions. The magnetisation measurements are extended to high fields for both HoB_4_ and NdB_4_. For NdB_4_ we also present field and temperature dependent resistivity measurements and construct the *H* − *T* magnetic phase diagrams for both $H\parallel c$ and $H\perp c$.

HoB_4_ shows successive phase transitions at ${T}_{{\rm{N1}}}^{{\rm{Ho}}}=7.1\,{\rm{K}}$ and ${T}_{{\rm{N2}}}^{{\rm{Ho}}}=5.7\,{\rm{K}}$, defining the intermediate temperature $({T}_{{\rm{N2}}}^{{\rm{Ho}}} < T < {T}_{{\rm{N1}}}^{{\rm{Ho}}})$ and low temperature $(T < {T}_{{\rm{N2}}}^{{\rm{Ho}}})$ magnetically ordered phases^20^. The magnetic structure for both phases has been determined by powder neutron diffraction^21^. The intermediate temperature phase has an incommensurate amplitude modulated magnetic order in which the moments are tilted from the *c* axis by approximately 25° towards the [110] direction. The order is characterised by the propagation vector (*δ*, *δ*, *δ*′), where *δ* = 0.022 and *δ*′ = 0.43. The low temperature phase is ordered with a non-collinear antiferromagnetic state.

Fractional magnetisation plateaux at $M/{M}_{{\rm{sat}}}=\tfrac{1}{3}$, with two less pronounced features at $M/{M}_{{\rm{sat}}}\approx \tfrac{4}{9}$ and $\tfrac{3}{5}$ have been observed in field dependent magnetisation measurements^20,22^. Single crystal neutron diffraction measurements have revealed the $\tfrac{1}{3}$-magnetisation plateau has a ferrimagnetic structure where ferromagnetic layers stack in an up-up-down configuration, with the moments pointing along the *c* axis^23^.

Heat capacity and magnetic susceptibility measurements on NdB_4_ show successive phase transitions at ${T}_{0}^{{\rm{Nd}}}=17\,{\rm{K}}$, ${T}_{{\rm{N1}}}^{{\rm{Nd}}}=7.0\,{\rm{K}}$ and ${T}_{{\rm{N2}}}^{{\rm{Nd}}}=4.8\,{\rm{K}}$ ^24^. The three transitions define the high $({T}_{{\rm{N1}}}^{{\rm{Nd}}} < T < {T}_{0}^{{\rm{Nd}}})$, intermediate $({T}_{{\rm{N2}}}^{{\rm{Nd}}} < T < {T}_{{\rm{N1}}}^{{\rm{Nd}}})$ and low $(T < {T}_{{\rm{N2}}}^{{\rm{Nd}}})$ temperature ordered phases in NdB_4_. These have been attributed to quadrupolar ordering, incommensurate and fully ordered phases respectively^24^. Although recently the commensurate magnetic structure in the high temperature phase has been determined to have an “all-in/all-out” antiferromagnetic order where the moments on four Nd ions in the unit cell are in the *ab* plane and point into the square formed by the Nd ions, cooling causes the the moments to rotate in the *ab* plane slightly. On the other hand the intermediate and low temperatures phases were found to be incommensurate^25^.

The structure of this report is as follows. The results and discussion is split into two subsections. The NdB_4_ subsection shows the magnetisation and resistivity measurements for NdB_4_ bringing all the measurements together in a magnetic phase diagram, while the next subsection shows the low-temperature and high-field magnetisation for HoB_4_. The next section summarises the findings. Finally the experimental details are described in the methods section.

## Results and Discussion

### NdB_4_

**Figure 2:**
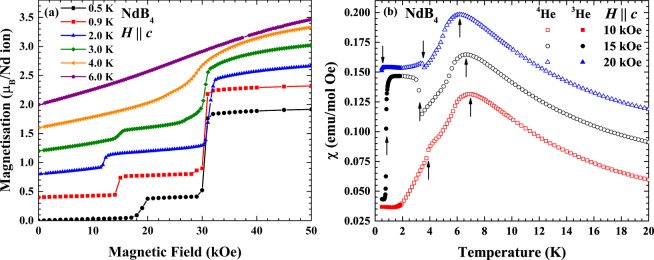
(**a**) Field dependent magnetisation curves of NdB_4_ for $H\parallel c$. Each curve is subsequently offset by 0.4 *μ*_B_ per Nd ion. (**b**) Temperature dependent magnetic susceptibility curves at 10, 15 and 20 kOe with $H\parallel c$. Open symbols correspond to measurements taken using ^4^He, while closed symbols correspond to those made using ^3^He and each curve is subsequently offset by 0.03 emu/mol-Oe. The arrows correspond to the position of the magnetic phase transitions.

Figure 2(a) shows the field dependent magnetisation curves for NdB_4_ single crystal samples. The temperature was varied between 0.5 and 6 K. For all temperatures below ${T}_{{\rm{N2}}}^{{\rm{Nd}}}=4.8\,{\rm{K}}$ the magnetisation curves indicate the presence of a plateau, which becomes more pronounced at lower temperatures. By *T* = 0.5 K the plateau is clearly defined by the lower and higher field transitions at *H* = 20 and 30 kOe respectively, the value of magnetisation on a plateau is approximately $\tfrac{1}{5}$ of the values observed in higher fields. While the transition at 30 kOe is almost temperature independent, the temperature dependence of the critical field of the transition to the plateau from lower fields appears to be non-monotonic. On warming from 0.5 K, the value of the transition decreases to 14 and to 12 kOe for 0.9 and 2 K respectively before increasing to 15 kOe at 3 K, with the plateau becoming less defined. While the transition to saturation at 3 K begins with a gradual up turn at approximately *H* = 26 kOe before a more significant jump at approximately 29 kOe. Increasing the temperature further to *T* = 4 K, the magnetisation plateau becomes significantly less pronounced and the transitions could only be seen in the derivatives of the magnetisation curve, d*M*/d*H* (not shown). For *T* = 4 K, the lower-field transition appears to be shift to 24 kOe, while the higher-field transition remains at approximately 30 kOe. The magnetisation curve at *T* = 6 K (in the intermediate temperature phase) shows no discernible transition, linearly increasing towards saturation.

The exact nature of the plateau phase is currently unknown, however, a $\tfrac{1}{5}$-magnetisation plateau is quite unusual as there are only four magnetic moments in the unit cell. Magnetisation plateaux with fractional values of $\tfrac{1}{7}$ and $\tfrac{1}{9}$ have been observed in TmB_4_^11^. Here neutron diffraction measurements revealed these phases had a striped structure consisting of antiferromagnetic regions 7 or 9 unit cells wide respectively, separated by a strip of ferromagnetic spins^11^. Alternatively the plateau arise from stacks of ferromagnetic layers along the *c* axis, similar to the proposed field-induced structure in HoB_4_^23^. Above *H* = 30 kOe, the magnetisation value is approximately 2 *μ*_B_ per Nd ion, remaining constant up to 100 kOe. The sample appears to have reached saturation, however, the saturated moment is significantly smaller compared to the expected saturated moment of 3.2 *μ*_B_ for Nd^3+^ ions.

To investigate this unusual temperature dependence of the transition field to the $\tfrac{1}{5}$ magnetisation plateau, magnetic susceptibility measurements at 10, 15 and 20 kOe were made, as shown in Fig. 2(b). The susceptibility curve at *H* = 10 kOe shows a broad maximum at *T* = 6.8 K and a discontinuous drop at *T* = 4.2 K. These correspond to transitions to the incommensurate intermediate and low temperature phases. Increasing the field to 15 kOe the broad maximum is suppressed to *T* = 6.7 K, while there is now a jump at *T* = 3.3 K, which is the magnetisation plateau state. Cooling further reveals a drop in the susceptibility at *T* = 0.8 K. Temperature dependent susceptibility at lower fields shows no indication of a new phase, suggesting the transition is a re-entrant phase transition occurring at *T* = 0.8 K where the field induced state returns to the zero-field antiferromagnetic state. Increasing the field to 20 kOe, ${T}_{{\rm{N1}}}^{{\rm{Nd}}}$ is further suppressed to 6.2 K, while ${T}_{{\rm{N2}}}^{{\rm{Nd}}}$ increases to 3.6 K. It can also be seen that the lower, novel transition is suppressed with the transition beginning at *T* ≈ 0.5 K, but we are unable to achieve lower temperatures to clearly see the entire transition.

**Figure 3:**
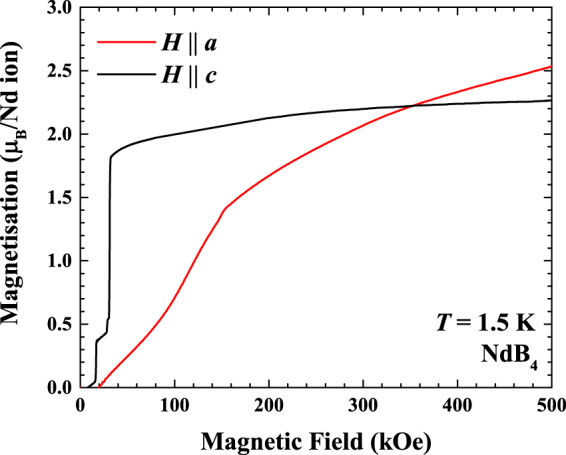
Field dependent magnetisation measured in NdB_4_ single crystal for $H\parallel c$ and $H\perp c$ using a pulsed magnetic field up to 500 kOe.

Figure 3 shows the field dependent magnetisation of NdB_4_ at *T* = 2 K for *H* $\parallel$ *c* and $H\perp c$ in a pulsed magnetic field up to 500 kOe. For $H\parallel c$, we observe the $\tfrac{1}{5}$ magnetisation plateau at 14 kOe and then the transition to saturation at 30 kOe. The magnetisation then increases only marginally from 2.0 *μ*_B_ per Nd ion in 100 kOe up to 2.3 *μ*_B_ in 500 kOe. The difference between the saturated moment and expected moment could be arising due to strong crystalline electric field effects^26^. For $H\perp c$ on the other hand, the magnetisation is linear up to approximately 70 kOe, with a gradual up turn in the value showing a distinct change in gradient at approximately 150 kOe. After the transition the magnetisation increases, surpassing the magnetisation of *H* $\parallel$ *c* at 350 kOe, the magnetisation continues to increase towards saturation, achieving 2.6 *μ*_B_ per Nd ion by 500 kOe. The difference between the two directions suggest the easy axis in high fields is along the *a* axis.

**Figure 4:**
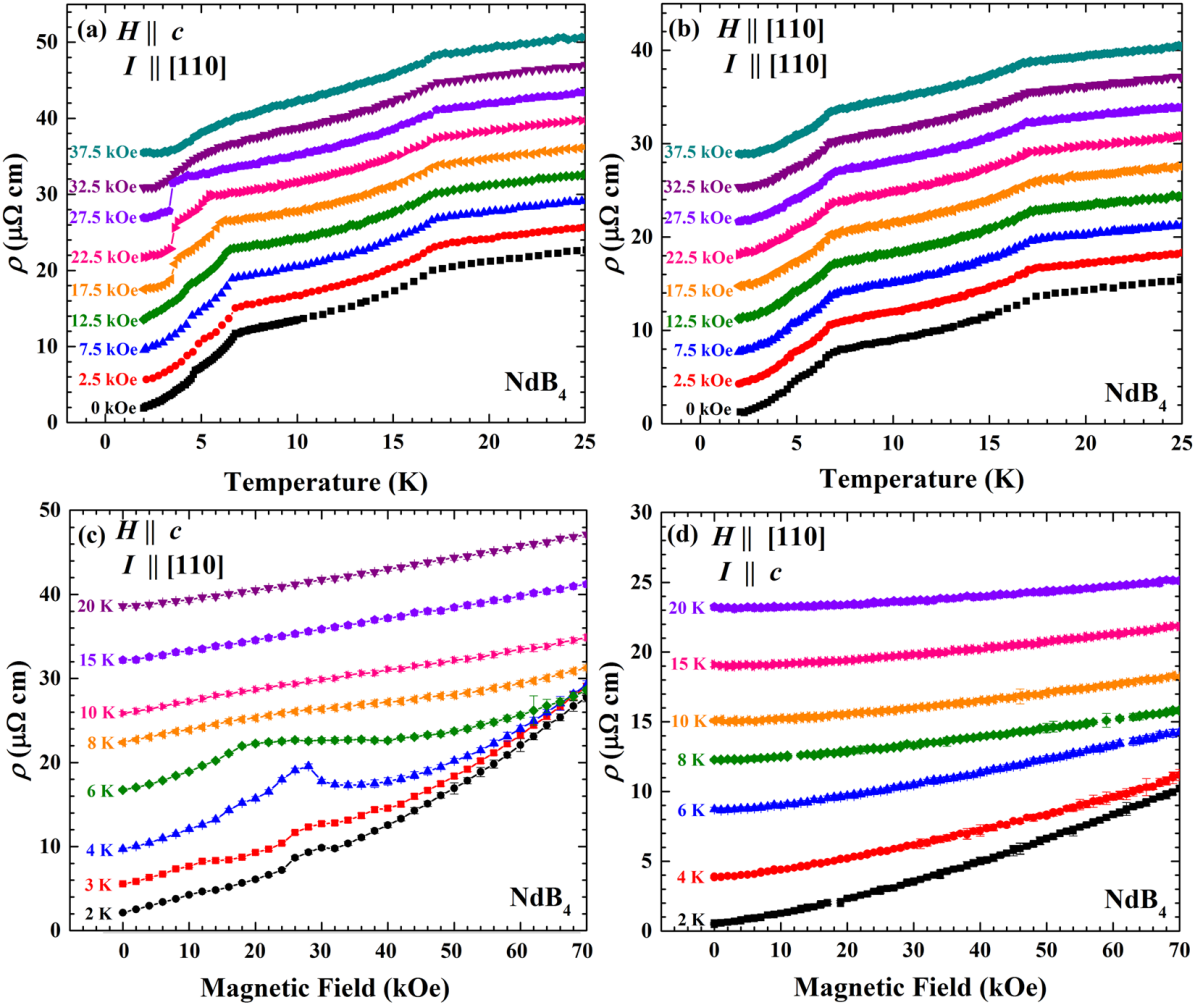
Temperature dependent resistivity measurements of NdB_4_ made for (**a**) $H\parallel c$ and (**b**) $H\perp c$. Each curve is subsequently offset by 2 and 3 *μ*Ω^−^cm respectively. Field dependent resistivity measurements made for (**c**) $H\parallel c$ and (d) $H\perp c$. Each curve is subsequently offset by 2.5 *μ*Ω^−^cm. For the panels (a–c) the current, *I*, is applied along the [110] direction, while the current is applied along the *c* axis in (**d**).

The temperature dependence of the resistivity of NdB_4_ single crystal samples is shown in Fig. 4 for both *H* $\parallel$ *c* and $H\perp c$. The NdB_4_ crystal studied has a high residual resistance ratio value (RRR; *ρ*(300 K)/*ρ*(2 K) = 100) suggesting a high quality sample. For both field directions the resistivity curves are linear in temperature down to the first transition, ${T}_{0}^{{\rm{Nd}}}$, indicating the metallic nature of NdB_4_ which is the case in the other *R*B_4_ family members. There is a sudden drop in the resistivity at ${T}_{0}^{{\rm{Nd}}}=17\,{\rm{K}}$, with two further features at ${T}_{{\rm{N1}}}^{{\rm{Nd}}}=6.8\,{\rm{K}}$ and ${T}_{{\rm{N2}}}^{{\rm{Nd}}}=4.9\,{\rm{K}}$. The sudden slope change could be due to the loss of spin disorder scattering at the antiferromagnetic transition temperature, which has been suggested for other members of the *R*B_4_ family^27,28^. The evolution of the temperature dependence of the resistivity in field for *H* $\parallel$ *c* is shown in Fig. 4(a). Similarly to previously published magnetic susceptibility curves^24^, the transitions at ${T}_{{\rm{N1}}}^{{\rm{Nd}}}$ and ${T}_{{\rm{N2}}}^{{\rm{Nd}}}$ are suppressed with increasing field. ${T}_{{\rm{N2}}}^{{\rm{Nd}}}$ becomes significantly more pronounced for *H* = 17.5, 22.5 and 27.5 kOe, which corresponds to the transitions into the $\tfrac{1}{5}$-magnetisation plateau phase. The transition becomes very broad above 27.5 kOe as the system tends towards a polarised state.

The temperature dependence of the resistivity for $H\perp c$ is shown in Fig. 4(b). We observe the three transitions at ${T}_{0}^{{\rm{Nd}}}$, ${T}_{{\rm{N}}1}^{{\rm{Nd}}}$ and ${T}_{{\rm{N}}2}^{{\rm{Nd}}}$, which do not change significantly between 0 ≤ *H* ≤ 37.5 kOe, mirroring the magnetic susceptibility and heat capacity measurements^24^.

The field dependent resistivity measurements for $H\parallel c$ are shown in Fig. 4(c). As can be seen there are large positive magnetoresistance (defined by MR(*H*, *T*) = [*ρ*(*H*, *T*) − *ρ*(0, *T*)]/*ρ*(0, *T*)) changes at all temperatures, the most significant of which is at *T* = 2 K with a value of MR(*H*, *T*) = 1200% for *H* = 70 kOe. Large magnetoresistences have also been observed in HoB_4_ with a value of 4000%^20^ and in GdB_4_ with a value of 58800%^29^. The resistivity curves at *T* = 2, 3 and 4 K show features which mirror the plateaux observed in the magnetisation measurements (see Fig. 2). The most pronounced feature is at 4 K, where there is a decrease in the resistivity at around 28 kOe. A plateau is also observed at *T* = 6 K, the constant resistivity most likely arising as the system is undergoing a transition from the intermediate to the high temperature phase. This response has also been observed in ErB_4_ and TmB_4_^30^, where the curves can be thought of as the sum of a conventional orbital magnetoresistance with additional scattering due to magnetic ordering in the plateau state.

The field dependent resistivity curves for $H\perp c$ are shown in Fig. 4(d). As can be seen there are no noticeable features at any temperature, consistent with the magnetisation measurements. Similarly to the $H\parallel c$ case, there is a magnetoresistence, which gradually increases with decreasing temperature. The most significant magnetoresistance is again at *T* = 2 K with a value of MR(*H*, *T*) = 2140% for *H* = 70 kOe.

**Figure 5:**
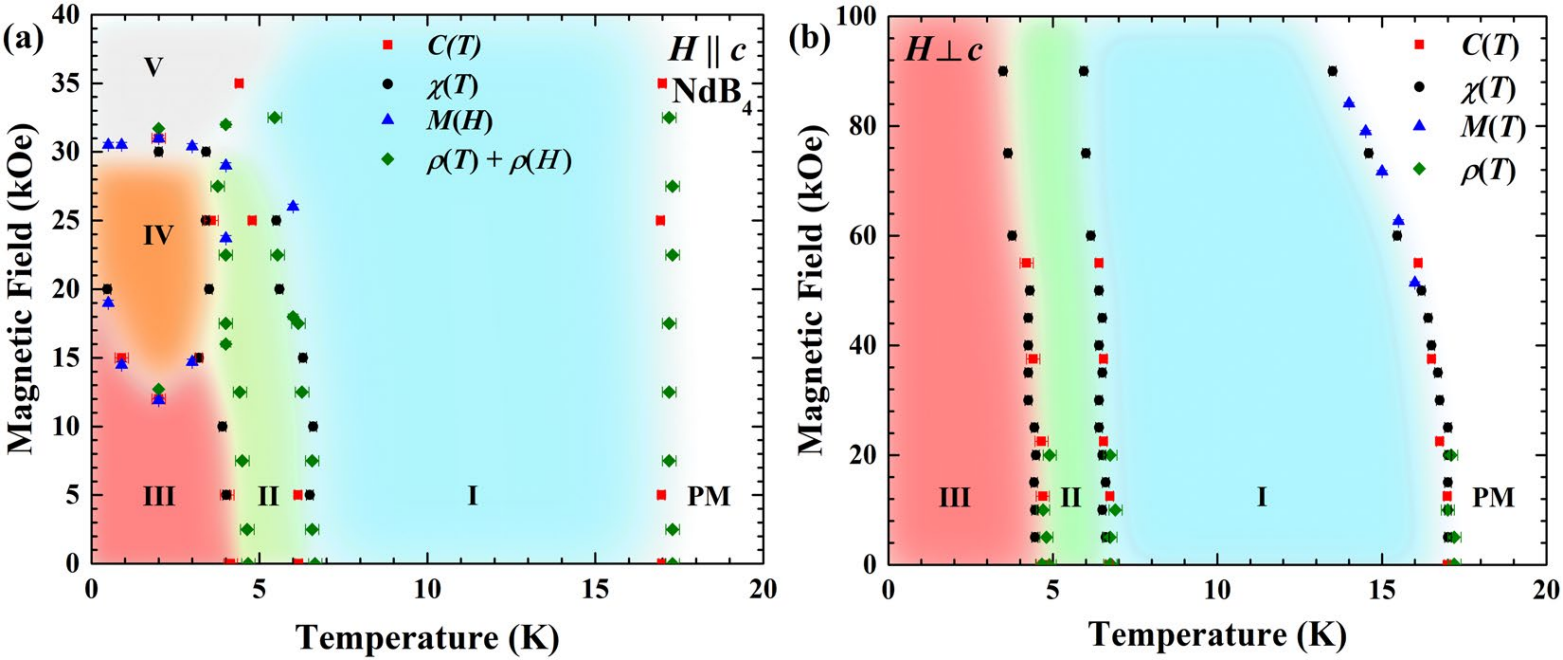
Magnetic phase diagrams of NdB_4_ for (**a**) $H\parallel c$ and (**b**) $H\perp c$. For both field directions, Phase I corresponds to a non-collinear antiferromagnetic order, while Phases II and III are two different incommensurate structures. Phases IV and V for $H\parallel c$ correspond to the $\tfrac{1}{5}$-magnetisation plateau and the fully polarised state respectively.

Figure 5 shows the magnetic phase diagrams of NdB_4_ constructed from magnetisation, heat capacity and resistivity measurements for $H\parallel c$ and $H\perp c$. Heat capacity measurements are not shown but are in agreement with previous published results^24^.

For $H\parallel c$, Fig. 5(a) the magnetic phase diagram contains five distinct magnetically ordered phases (labelled with Roman numerals I to V) as well as a paramagnetic (PM) phase. The high-temperature Phase I remains largely unaffected by application of a magnetic field. The magnetisation curves suggest no sign of saturation up to 100 kOe. The incommensurate intermediate temperature phase (Phase II) seems to disappear in fields above 30 kOe. The low-temperature zero-field state (Phase III) transforms to Phases IV and V in an applied field with the unusually shaped magnetic transition line between phases III and IV showing a clear minimum at approximately 2 K. Phase V corresponds to the fully polarised phase.

For $H\perp c$, the phase diagram (Fig. 5(b)) contains three distinct ordered states (labelled with Roman numerals I to III) as well as the PM phase. Apart from a slight reduction of the transition temperatures (particularly for the PM to the phase I transition) on application of magnetic field, all three ordered phases remain largely unaffected.

### HoB_4_

**Figure 6:**
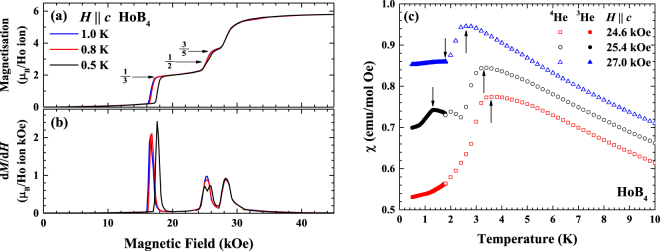
(**a**) Field dependent magnetisation and (**b**) its derivative d*M*/d*H* of HoB_4_ for temperatures below 1 K. The derivative curves highlight field-induced transitions. The magnetisation curves were recorded for both increasing and decreasing fields, but, for clarity, only those corresponding to the increasing field are shown. (**c**) Temperature dependent magnetic susceptibility at *H* = 24.6, 25.4 and 27.0 kOe. Each curve is subsequently offset by 0.05 emu/mol-Oe. The arrows correspond to the position of the magnetic phase transitions.

Figure 6(a) shows the field dependent magnetisation of HoB_4_ for 0.5, 0.8 and 1 K. The magnetisation curve at *T* = 1 K shows a magnetisation plateau at $M/{M}_{{\rm{sat}}}=\tfrac{1}{3}$ and a less pronounced feature at $M/{M}_{{\rm{sat}}}\approx \tfrac{3}{5}$, which is consistent with previous observations at *T* = 2 K^20,22^. An additional transition has been observed previously at $M/{M}_{{\rm{sat}}}\approx \tfrac{4}{9}$ ^23^, this was found to depend on the exact orientation of the crystal with the field. When the *c* axis was slightly tilted from the field, the transition is visible, while when the field is aligned along the *c* axis it is no longer visible. With temperature decreasing to 0.5 K, the feature at $M/{M}_{{\rm{sat}}}\approx \tfrac{3}{5}$ becomes more pronounced while the transition from zero-field structure to the plateau shifts to higher applied fields. The magnetisation curve at *T* = 0.5 K shows clear magnetisation plateaux at $M/{M}_{{\rm{sat}}}=\tfrac{1}{3}$ and $\tfrac{3}{5}$, while there is an additional transition occurring at $M/{M}_{{\rm{sat}}}\approx \tfrac{1}{2}$. This extra feature is highlighted in the differentiated magnetisation curves, d*M*/d*H*, shown in Fig. 6(b), where the $M/{M}_{{\rm{sat}}}=\tfrac{1}{2}$ state is seen as the splitting of the higher-temperature peak of the derivative into two close and partially overlapping peaks around 25 kOe at *T* = 0.5 K. Interestingly, neutron diffraction measurements^23^ revealed that the $\tfrac{1}{3}$-magnetisation plateau is associated with an up-up-down ferrimagnetic stacking along the *c* axis. Therefore a two-dimensional SSL model is not well suited for describing the $M/{M}_{{\rm{sat}}}=\tfrac{1}{3}$ plateau. It is, however, possible that the SSL physics is applicable to this new $\tfrac{1}{2}$ magnetisation feature.

To investigate this new field-induced state, the temperature dependent magnetic susceptibility measurements were made in a magnetic field of 25.4 kOe corresponding to the local minimum between the two magnetisation jumps in the d*M*/d*H* for *T* = 0.5 K, as well as in fields just above and below it (see Fig. 6(c)). All three susceptibility curves show a broad maximum at around 3.7 K, which is slightly shifted to lower temperatures by an increasing magnetic field. At *H* = 25.4 kOe, a clear extra feature is observed in *χ*(*T*) at *T* ≈ 1 K, which is not seen at either *H* = 24.6 kOe or *H* = 27.0 kOe. This observation provides additional support to the claim that $M/{M}_{{\rm{sat}}}=\tfrac{1}{2}$ might be stabilised in HoB_4_ in a narrow field interval at a sufficiently low temperature.

**Figure 7:**
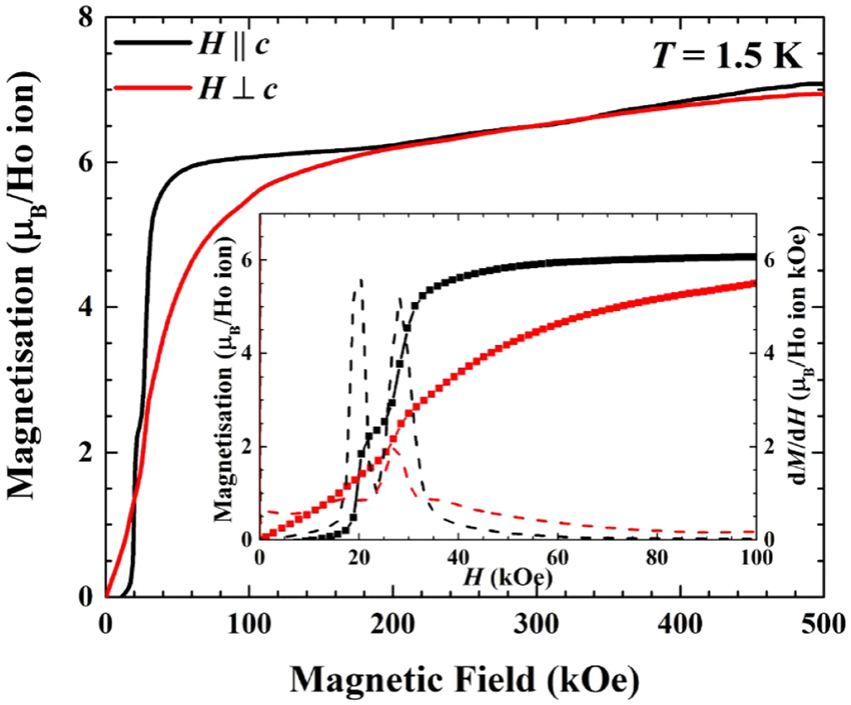
Field dependent magnetisation measurements of HoB_4_ for $H\parallel c$ and $H\perp c$ using a pulsed magnetic field up to 500 kOe. Inset highlights the fractional magnetisation plateaux at low magnetic fields by showing the magnetisation (symbols) and its derivative d*M*/d*H* (dashed lines).

Figure 7 shows the field dependent magnetisation of HoB_4_ in fields up to 500 kOe for both $H\parallel c$ and $H\perp c$. The inset shows the magnetisation curves and derivative in a smaller field range to highlight the transitions observed, which are in agreement with previous measurements^20^. As can be seen the magnetisation initially levels off at 6 *μ*_B_ per Ho ion in a field of approximately 40 kOe and then gradually increases up to approximately 7 *μ*_B_ per Ho ion. For $H\perp c$, the behaviour is very different in lower fields, but above 200 kOe the magnetisation curves for $H\parallel c$ and $H\perp c$ are practically indistinguishable. During the experiment we have found that upon application of the magnetic field the sample experienced significant torque and had a tendency to deviate away from the $H\parallel c$ and $H\perp c$ geometries unless firmly attached to a sample holder. These observations suggest that the *c* axis may not be the easy magnetisation direction in HoB_4_ in higher applied fields.

## Summary

We have made detailed investigation of the magnetisation process in NdB_4_ and HoB_4_ single crystal samples at low temperatures.

For $H\parallel c$, the dominant feature in magnetisation of NdB_4_ is the $\tfrac{1}{5}$-magnetisation plateau. Our measurements have revealed rather unusual non-monotonic behaviour of critical fields in NdB_4_ where in an applied field of around 15 kOe low temperatures see the field induced magnetic phase be replaced by the zero field structure. For $H\perp c$, high field scans have revealed a broad transition at around 70 kOe and a clear slope change in magnetisation at 150 kOe. Resistivity measurements mirror the transitions observed in magnetisation and heat capacity, they also lead to the observation of a large magnetoresistance for various field directions. Large anisotropy of the magnetic properties of NdB_4_ is emphasised by the different shape of the reconstructed *H* − *T* magnetic phase diagrams for $H\parallel c$ and $H\perp c$.

Magnetisation measurements on HoB_4_ have revealed the stabilisation of a $\tfrac{1}{2}$ plateau, which arises below *T* ≈ 1 K for a narrow field range in addition to the $\tfrac{1}{3}$ and $\tfrac{3}{5}$ plateaux also seen at higher temperatures. The $\tfrac{1}{3}$-magnetisation plateau in HoB_4_ cannot be described within the SSL model^23^, however, the applicability of SSL model to the $\tfrac{1}{2}$ plateau is still to be tested. It would be interesting to extend in-field neutron diffraction measurements to sufficiently low temperatures to thoroughly investigate the nature of this new phase. Other *R*B_4_ members, such as ErB_4_ and TmB_4_^11,30^ show a more defined $\tfrac{1}{2}$ fractional magnetisation plateau and are well described by the SSL, with the proposed structures centring around antiferromagnetic and ferromagnetic stripes.

The high field measurements on HoB_4_ show no further transitions, where the magnetisation curve initially levels off, before there is a slight upturn at 190 kOe that matches the curve where $H\perp c$. Both curves gradually increase, reaching 7 *μ*_B_/Ho ion at 500 kOe.

## Methods

Polycrystalline rods of HoB_4_ and NdB_4_ were prepared by arc melting the constituent elements in an argon atmosphere and the floating zone technique using an image furnace with four Xenon-arc lamps was then utilised to grow single crystals. The crystals were checked and aligned using a backscattering x-ray Laue system.

A Quantum Design SQUID magnetometer was used to measure magnetic susceptibility and magnetisation along each of the principal axes between 1.8 < *T* < 300 K and 0 < *H* < 70 kOe. The temperature range was extended to 0.5 K using an iQuantum ^3^He low temperature insert^31^. Additional field scans up to 100 kOe were made using an Oxford Instruments vibrating sample magnetometer (VSM) down to temperatures of 1.45 K. High field magnetisation measurements were made using a pulsed magnetic field up to 580 kOe at the Dresden High Magnetic Field Laboratory (HLD), Germany. The measurements were carried out on small (typically less than 10 mg) plate-like samples aligned along a principal axis, such that the field was parallel to the plate face to minimise the demagnetising factor. The demagnetising factor did not exceed a value of 0.22 and therefore the correction for the effective field did not exceed 1% at the transition fields. The demagnetisation factor was determined by following the process outlined by Aharoni^32^. This geometry was used for both $H\parallel c$ and $H\perp c$. AC resistivity measurements were made using the 4-probe method between 1.8 and 300 K in magnetic fields up to 70 kOe using a Quantum Design Physical Property Measurement System. For all resistivity measurements a current, *I*, of 10 mA and a frequency of 113 Hz was used with the current applied perpendicular to the field direction.

## References

[1] Collins MF, Petrenko OA (1997). Review/synthèse: Triangular antiferromagnets. Can. J. Phys..

[2] Gardner JS, Gingras MJP, Greedan JE (2010). Magnetic pyrochlore oxides. Rev. Mod. Phys..

[3] Ramirez AP (1994). Strongly geometrically frustrated magnets. Annu. Rev. Mater. Sci..

[4] Bramwell ST, Gingras MJP (2001). Spin ice state in frustrated magnetic pyrochlore materials. Sci..

[5] Schiffer P, Ramirez AP, Huse DA, Valentino AJ (1994). Investigation of the field induced antiferromagnetic phase transition in the frustrated magnet: Gadolinium gallium garnet. Phys. Rev. Lett..

[6] Chalker JT, Eastmond JFG (1992). Ground-state disorder in the spin-1/2 kagomé heisenberg antiferromagnet. Phys. Rev. B.

[7] Miyahara S, Ueda K (2003). Theory of the orthogonal dimer heisenberg spin model for srcu2 (bo_3_)_2_. J. Physics: Condens. Matter.

[8] Shastry BS, Sutherland B (1981). Exact ground state of a quantum mechanical antiferromagnet. Phys. B + C.

[9] Buschow KHJ, Creyghton JHN (1972). Magnetic properties of rare earth tetraborides. The J. Chem. Phys..

[10] Pfeiffer F, Schäfer W, Will G, Etourneau J, Georges R (1979). The magnetic phase diagram of ErB_4_. J. Magn. Magn. Mater..

[11] Siemensmeyer K (2008). Fractional magnetization plateaus and magnetic order in the shastry-sutherland magnet TmB_4_. Phys. Rev. Lett..

[12] Yoshii S (2008). Multistep magnetization plateaus in the shastry-sutherland system TbB_4_. Phys. Rev. Lett..

[13] Yoshii S (2006). High-field magnetization of TmB_4_. Journal of Physics: Conf. Ser..

[14] Moliner, M., Cabra, D. C., Honecker, A., Pujol, P. & Stauffer, F. Magnetization process in the classical Heisenberg model on the Shastry-Sutherland lattice. *Phys. Rev. B* **79**, 144401 (2009).

[15] Huo, L. *et al*. The competing spin orders and fractional magnetization plateaus of the classical Heisenberg model on Shastry-Sutherland lattice: Consequence of long-range interactions. *J*. *Appl*. *Phys*. **113**, 073908 (2013).

[16] Huang, W. C. *et al*. Multi-step magnetization of the Ising model on a Shastry–Sutherland lattice: a Monte Carlo simulation. *J. Physics: Condens. Matter* **24**, 386003 (2012). 10.1088/0953-8984/24/38/386003 22927561

[17] Dublenych YI (2013). Ground states of an ising model on an extended shastry-sutherland lattice and the 1/2-magnetization plateau in some rare-earth-metal tetraborides. Phys. Rev. E.

[18] Grechnev A (2013). Exact ground state of the shastry-sutherland lattice with classical heisenberg spins. Phys. Rev. B.

[19] Feng, J. J. *et al*. The main 1/2 magnetization plateau in Shastry-Sutherland magnets: Effect of the long-range Ruderman-Kittel-Kasuya-Yosida interaction. *EPL* (*Europhysics Lett*. **105**, 17009 (2014).

[20] Kim JY, Cho BK, Han SH (2009). Anisotropic magnetic phase diagrams of HoB_4_ single crystal. J. Appl. Phys..

[21] Okuyama, D. *et al*. Competition of magnetic and quadrupolar order parameters in HoB_4_. *J. Phys. Soc. Jpn.* **77**, 044709 (2008).

[22] Mat’as S (2010). Magnetism of rare earth tetraborides. J. Physics: Conf. Ser..

[23] Brunt D (2017). Field-induced magnetic states in holmium tetraboride. Phys. Rev. B.

[24] Watanuki R, Kobayashi T, Noguchi R, Suzuki K (2009). Possible multipolar transition in NdB_4_. J. Physics: Conf. Ser..

[25] Yamauchi H (2017). Magnetic structure and quadrupolar order parameter driven by geometrical frustration effect in NdB_4_. J. Phys. Soc. Jpn..

[26] Jensen, J. & Mackintosh, A. R. *Rare Earth Magnetism Structures and Excitations* (Clarendon Press Oxford, 1991).

[27] Kim JY (2010). Magnetic anisotropy and magnon gap state of SmB_4_ single crystal. J. Appl. Phys..

[28] Rhyee, J.-S., Kim, J. Y. & Cho, B. K. Multiple magnetic transitions and magnon gaplike characteristics in the high purity TbB_4_ single crystal. *J*. *Appl*. *Phys*. **101**, 09D509 (2007).

[29] Cho, B. K., Rhyee, J.-S., Kim, J. Y., Emilia, M. & Canfield, P. C. Anomalous magnetoresistance at low temperatures (*T* = 10 K) in a single crystal of GdB_4_. *J. Appl. Phys.* **97**, 10A923 (2005).

[30] Ye L, Suzuki T, Checkelsky JG (2017). Electronic transport on the shastry-sutherland lattice in ising-type rare-earth tetraborides. Phys. Rev. B.

[31] Shirakawa, N., Horinouchi, H. & Yoshida, Y. Measuring Sr2RuO4 down to 0.5 K with a commercial {SQUID} magnetometer combined with ^3^he refrigeration. Proceedings of the International Conference on Magnetism (ICM 2003). *J*. *Magn*. *Magn*. *Mater*. **27**2**–276**, Supplement, E149–E150 (2004).

[32] Aharoni A (1998). Demagnetizing factors for rectangular ferromagnetic prisms. J. Appl. Phys..

